# Modelling the Abundances of Two Major *Culicoides* (Diptera: Ceratopogonidae) Species in the Niayes Area of Senegal

**DOI:** 10.1371/journal.pone.0131021

**Published:** 2015-06-29

**Authors:** Maryam Diarra, Moussa Fall, Renaud Lancelot, Aliou Diop, Assane G. Fall, Ahmadou Dicko, Momar Talla Seck, Claire Garros, Xavier Allène, Ignace Rakotoarivony, Mame Thierno Bakhoum, Jérémy Bouyer, Hélène Guis

**Affiliations:** 1 Institut Sénégalais de Recherches Agricoles, Laboratoire National de l’Elevage et de Recherches Vétérinaires, Dakar, Sénégal; 2 Université Gaston Berger, Laboratoire d’Etudes et de Recherches en Statistiques et Développement, Saint-Louis, Sénégal; 3 Cirad, UMR15 CMAEE, F-34398 Montpellier, France; 4 INRA, UMR1309 CMAEE, F-34398 Montpellier, France; University of Minnesota, UNITED STATES

## Abstract

In Senegal, considerable mortality in the equine population and hence major economic losses were caused by the African horse sickness (AHS) epizootic in 2007. *Culicoides oxystoma* and *Culicoides imicola*, known or suspected of being vectors of bluetongue and AHS viruses are two predominant species in the vicinity of horses and are present all year-round in Niayes area, Senegal. The aim of this study was to better understand the environmental and climatic drivers of the dynamics of these two species. *Culicoides* collections were obtained using OVI (Onderstepoort Veterinary Institute) light traps at each of the 5 sites for three nights of consecutive collection per month over one year. Cross Correlation Map analysis was performed to determine the time-lags for which environmental variables and abundance data were the most correlated. *C*. *oxystoma* and *C*. *imicola* count data were highly variable and overdispersed. Despite modelling large *Culicoides* counts (over 220,000 *Culicoides* captured in 354 night-traps), using on-site climate measures, overdispersion persisted in Poisson, negative binomial, Poisson regression mixed-effect with random effect at the site of capture models. The only model able to take into account overdispersion was the Poisson regression mixed-effect model with nested random effects at the site and date of capture levels. According to this model, meteorological variables that contribute to explaining the dynamics of *C*. *oxystoma* and *C*. *imicola* abundances were: mean temperature and relative humidity of the capture day, mean humidity between 21 and 19 days prior a capture event, density of ruminants, percentage cover of water bodies within a 2 km radius and interaction between temperature and humidity for *C*. *oxystoma*; mean rainfall and NDVI of the capture day and percentage cover of water bodies for *C*. *imicola*. Other variables such as soil moisture, wind speed, degree days, land cover or landscape metrics could be tested to improve the models. Further work should also assess whether other trapping methods such as host-baited traps help reduce overdispersion.

## Introduction

Bluetongue (BT) and African horse sickness (AHS) are two devastating *Culicoides*-borne viral diseases affecting ruminants and equids respectively. The importance of these two arboviral diseases derives from their very broad geographical distribution, their potential for spreading rapidly and their major economic impact, justifying their status of notifiable diseases by the World Animal Health Organisation [1]. Both viruses are transmitted by females of several species of midges belonging to the large *Culicoides* (Diptera: Ceratopogonidae) genus (which accounts for more than 1,300 species described worldwide [2, 3]).

In Senegal, the last epidemic of AHS occurred in 2007. It caused considerable economic loss, estimated to 0.9 billion FCFA (Franc des Communautés Financières d'Afrique, 1.4 million euros) [4]. On a national scale, this epizootic led to an estimated mortality rate of 0.2% and a morbidity rate of 0.3% in traditional horse breeding farms, and a 5.4% mortality rate in modern farms [4, 5]. In the Niayes area, particularly affected by the outbreak, several studies were initiated in order to better understand *Culicoides* diversity and their role in the AHS and BT viruses transmission. Results showed that *Culicoides oxystoma* (43.33% of the total capture) and *Culicoides imicola* (12.5% of the total capture) were the predominant species in the vicinity of horses and were present all the year [6]. It was the first record of *Culicoides oxystoma* in the Afrotropical region [7]. This species is a suspected bluetongue virus (BTV) vector in Australasia and Oceania [8, 9]. *Culicoides imicola* is known to be a vector of BTV [10–12] and African horse sickness virus (AHSV) [11, 13, 14]. There are many environmental and climatic variables associated with *Culicoides* [15]. For *C*. *imicola* in particular; temperature [16–23], rainfall [22–24], wind speed [24], altitude [17, 20, 21, 25, 26], NDVI (normalized difference vegetation index) [16, 17, 21, 23, 24, 27, 28], MIR (middle infrared reflectance) [17, 22, 23], host densities [28] and sparse vegetation habitat [21] were associated with the presence and abundance of this species. These drivers relate to the distribution of *C*. *imicola* in Europe mainly [18–23, 25, 28]. In Africa, drivers of its distribution have only been studied in Morocco [17, 24, 27] and in South Africa [16]. To our knowledge, there are no published models for *C*. *oxystoma*. The aim of the current study was to better understand the environmental and climatic drivers of the dynamics of *Culicoides* species important in terms of abundance in the vicinity of horses in the Niayes area and known or suspected of being vectors of BTV or AHSV, namely *C*. *oxystoma* and *C*. *imicola*.

One of the most commonly used statistical distribution to describe count data is the Poisson distribution that assumes equidispersion of the counts. However, in real datasets, these counts are often overdispersed [29–31] and various methods can be used to demonstrate it [32, 33]. The causes of overdispersion include the excess of zeros and/or missing covariates or interactions [34]. Ways to deal with count overdispersion include the use of other types of models such as negative binomial (NB) models or adding random effects at the site of capture and/or at the date of capture in a Poisson regression mixed-effect model [34]. In the present work, we assessed the ability of Poisson, NB, Poisson regression mixed-effect model with random effects at the site of capture (PRME-S) and with random effects at the site and date of capture (PRME-SD) to fit the distribution of counts of *C*. *oxystoma* and *C*. *imicola* measured monthly in 5 sites in Niayes area of Senegal during one year.

## Methods

### Vector monitoring

Data collected in five horse stables (Mbao (longitude: -17.3327; latitude: 14.7467), Parc Hann ((longitude: -17.4298; latitude: 14.7283)), Pout (longitude: -17.0357; latitude: 14.7665), Thies (longitude: -16.95; 14.794) and Niague (longitude: -17.2499; latitude: 14.8234)) in the Niayes area of Senegal from July 2011 to June 2012 were used in this study. Monitoring *Culicoides* midge apparent abundance over time was conducted by capturing the insects for three consecutive nights every month at each site over a period of one year. Two OVI (Onderstepoort Veterinary Institute) light traps were placed at each site at a distance of more than 10 m from each other. Indeed, the use of more than one trap per site allows for a more accurate sampling scheme as demonstrated in [35]. They were operated in the evening before dusk and collected the following morning after dawn. The traps were positioned close to the horses (hung on a post or a tree in the vicinity of the animals) at a height of 1.5 to 2 m from the ground. The identification of *Culicoides* was explained by Diarra et al. [6] and for more detailed information see [36]. Abundances from the two traps were summed for each night and each site. The maximum abundance of the sum of the two traps for three nights of collection per month and per site was used in model building. Maximum catches were preferred to mean catches because the number of specimens caught in a trap can drop very quickly when local weather conditions are sub-optimal [37]. Hence, the maximum of several consecutive catches was considered as the best measure for abundance over a short time period [37].

*Culicoides Oxystoma* and *Culicoides imicola* database are provided as supporting information (S1 and S2 Data).

### Meteorological and environmental data

This study included a total of 10 variables belonging to the following categories: land cover, livestock density, vegetation index (mean covering the catch event and interval lagged NDVI), temperature (mean of the catch day and interval lagged temperature), humidity (mean of the catch day and interval lagged humidity) and precipitation (mean of the catch day and interval lagged precipitation). These variables were extracted in a 2 km radius around the trap sites because *Culicoides* spp. can easily spread as far as 2 km from their breeding sites [38]. The land cover information was derived from FAO dataset (http://www.fao.org/geonetwork/srv/en/main.home?uuid=545be438-bc87-480b-83ec-ba3f4e486daf). The class ‘water bodies’ was selected because water is known to be an important characteristic of *Culicoides* breeding sites for both *C*. *imicola* [20, 26, 39–41] and *C*. *oxystoma* [42, 43]. *Culicoides imicola* larvae can be found in breeding sites composed of a rich mixture of organic matter and damp soil [39]. Such areas with wet organic soil can be found in many areas on a farm: in irrigated areas, areas with leaky taps or drinking troughs, or other small sources of water. *Culicoides oxystoma* larvae have been found in different breeding sites: sandflats and intertidal mud [42, 44], in paddy fields, pond margins and stream edges [45]. The percentage cover of water bodies within 2 km radius was computed for each site. Livestock density influences abundance of some species of *Culicoides* as it was shown for *C*. *obsoletus* [46] and *C*. *imicola* [47]. Livestock density (cattle, goats and sheep) was obtained from FAO dataset (http://www.fao.org/ag/againfo/resources/en/glw/GLW_dens.html). Mean density of domestic ruminants (sum (cattle+goats+sheep)/3) within 2 x 2 km from each site was calculated. The NOAA (National Oceanic and Atmospheric Administration) data for daily rainfall were extracted for each of the 5 sampling sites, (http://iridl.ldeo.columbia.edu/expert/SOURCES/.NOAA/.NCEP/.CPC/.FEWS/.Africa/.DAILY/.ARC2/.daily/.est_prcp/). The 10-day normalized difference vegetation index (NDVI) data was obtained from Spot-Vegetation (https://rs.vito.be/africa/en/data/Pages/vegetation.aspx). Daily temperature and relative humidity were obtained from Hobo data logger (http://www.microdaq.com/occ/u10/u10-003.php) set up at each site.

Missing data of temperature and relative humidity were estimated by Multivariate Singular Spectrum Analysis (MSSA) [48–50]. Statistical descriptions of the MSSA analysis are provided as supporting information (see S1 Text). This method was evaluated on meteorological data by calculating the mean absolute prediction error (MAPE) defined as:

$$MAPE=\frac{1}{N}\sum_{i=1}^{N}\frac{\left|obs_{i}-pred_{i}\right|}{obs_{i}}$$ (1)

With *obs* the meteorological records in the field and *pred* the MSSA estimations.

### Visualizing time-dependant associations

Meteorological variables such as temperature, humidity and rainfall affect different development stages of *Culicoides* life cycle, but precise quantitative assessments of the timing and importance of these relations and interactions for each life stage are lacking. Thus, to determine the time lags for which each variable (temperature, relative humidity, rainfall and NDVI) was the most correlated to *Culicoides* counts, we performed a Cross Correlation Map (CCM) analysis. CCM has been used to determine the time lags for which correlations between environmental variables and the abundance of a wide range of vectors such as mosquitoes [51–54] and *Culicoides* [55] are maximal.

Using a more mathematical notation, the statistical analysis reads as follows:

For *Y*_*i*_ representing the number of captured *Culicoides* at time *i* and *X*_*i*−*j*,*i*−*k*_ a meteorological quantity, averaged over a time period starting at time *i* − *j* (time lag 1) and ending at *i* − *k* (time lag 2), with *j* ≥ *k*, a CCM at the coordinates *j* and *k* illustrates:

$$CCM_{j,k}=cor(Y_{i},X_{i-j,\;i-k})$$ (2)

For the special case of *j* = *k* (plotted in the diagonal), the correlation coefficients are equal to those of a cross-correlogram.

For each species, a CCM was performed using time frames of 30 days for temperature, relative humidity and rainfall, and a time frame of 90 days for NDVI.

### Modelling vector abundance

Environmental covariates included livestock density, land cover, daily temperature (T), relative humidity (H), rainfall (R) (corresponding to the catch day), 10-day NDVI (covering the catch event) and the interval lagged T, H, R and NDVI. For each species, a univariate analysis was performed to visualize the relationship between the species abundance and each of the covariates, but also to assess the correlation between different covariables. Variables found to be highly correlated (coefficient of correlation greater than 0.5) with others covariables (provided as supporting information S1 and S2 Tables) were not kept for the multivariate analysis. Two interaction terms between temperature and humidity and between temperature and rainfall were introduced to the model as these variables are often found to influence life cycles in interaction with each other.

*C*. *oxystoma* and *C*. *imicola* count data were assessed according to a set of environmental covariates using 4 different models: a Poisson regression (P), a negative binomial (NB), a Poisson regression mixed-effect model with random effect at the site of capture (PRME-S) and a Poisson regression mixed-effect model with nested random effects at the site and date of capture levels (PRME-SD).The four models were evaluated to see whether they accounted for overdispersion (the overdispersion test used is well described in [56]). The database was divided into two datasets. We randomly chose 2/3 of the sample for the training dataset with which the coefficients of the models were estimated, and assessed the predictions on the remaining 1/3 of the sample, the test dataset. The root mean square error (RMSE) was calculated in the training (internal validation) and test datasets (external validation). The RMSE is defined as:

$$RMSE=\sqrt{\frac{1}{\text{N}}\sum_{\text{i}=1}^{\text{N}}{\left|{\text{Y}}_{\text{i}}-{\hat{Y}}_{i}\right|}^{2}}$$ (3)

For the *Culicoides* abundance data *Y*_*ij*_ counted on site *i* and date *j*, which takes any non-negative integer as its value, for *i* =1,…, *p* (p = 5 (5 sites)) and *j* = 1,…, *q* (q = 12 (12 dates)) as *y*_*ij*_:

- The stochastic component is described by a Poisson distribution with parameter *λ*_*ij*_

$$Y_{ij}\;∼Poisson\;\left(y_{ij}|\lambda_{ij}\right)=\frac{\lambda_{ij}{}^{y_{ij}}\text{exp}(-\lambda_{ij})}{y_{ij}!}$$ (4)

Where *y*_*ij*_ = 0,1,…

- The 2-dimensional vector of random effects (site and date), *b*_*i*_, is restricted to be of mean zero, and therefore is completely characterized by the variance covariance matrix *φ*, a (2×2) symmetric positive matrix. *b*_*i*_ ∼ *Normal* (0, *φ*)
- The systematic component is:

$$log\left(y_{ij}\right)=\beta^{T}\;{\text{X}}_{ij}+b_{i}\;Z_{ij}$$ (5)

Where *X*_*ij*_ is the (*p*×*q*×*M*) array of known fixed effects explanatory variables, *β* is the *M*-dimensional vector of fixed effects coefficients, *Z*_*ij*_ is the (*p*×*q*×2) array of known random effects explanatory variables and *b*_*i*_, the 2-dimensional vector of random effects (site and date) [57].

Parameter estimation was done by maximum likelihood [58]. A backward procedure was applied to select the variables that remained significant with a p-value < 0.05 in the final model. All analyses were performed using the ‘R’ software [59], with ‘lme4’ (for the model) and ‘Rssa’ (for gap filling temperature and relative humidity data) packages.

### Ethics Statements

This sampling protocol was approved by the Scientific and Technological Advice board of ISRA (Institut Sénégalais de Recherches Agricoles), Senegal on 22–27 November 2010. The owners of farms gave permission to conduct the study on their sites. Field workers did not have any contact with the horses.

## Results

The 354 night-traps captured 78,069 females of *C*. *oxystoma* and 24,176 females of *C*. *imicola* in 4 sites (Mbao, Pout, Thies and Parc Hann) from July 2011 to June 2012 and from November 2011 to October 2012 in Niague. The total of maximum monthly abundance was 30,070 for *C*. *oxystoma* and 13,904 for *C*. *imicola*.

**Fig 1:**
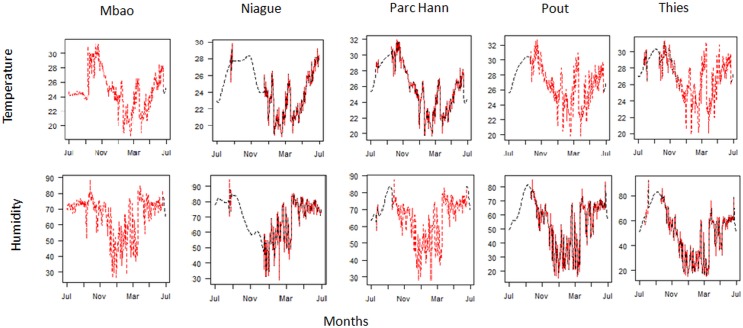
Gap filing data using Multivariate Singular Spectrum Analysis (MSSA). Estimating temperature and relative humidity missing data using MSSA method in each study site. In red the recorded environmental quantities, in black the reconstructed environmental quantities using MSSA method.

**Fig 2:**
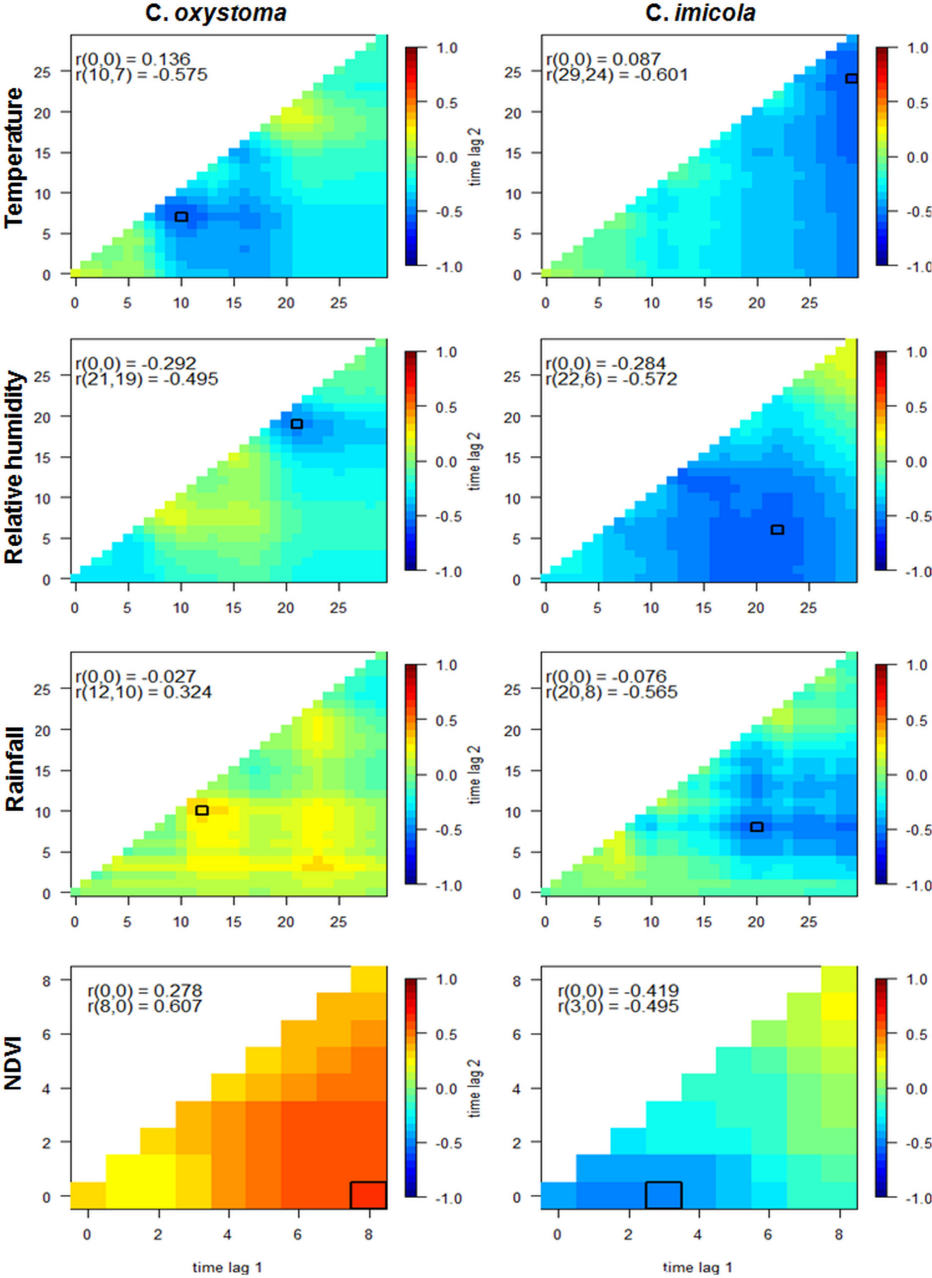
Cross Correlation Maps. Cross correlation maps for female *C*. *oxystoma* and *C*. *imicola* abundance with temperature, relative humidity, rainfall and NDVI

As missing data occurred mainly at the end of the rainy season, when catches were at their maximum [6], it was essential to estimate their values. Results from MSSA method used to fill the gaps in temperature and relative humidity data are presented in Fig 1. Missing data of temperature and relative humidity were present in each site: at Mbao, they were 3% of missing data, Niague 43.98%, Parc Hann 22.95%, Pout 24.31% and Thies 20.49%. Overall, in the 5 sites, prediction errors were low: for temperature MAPE = 0.07 and for relative humidity MAPE = 0.08. *C*. *oxystoma* abundances were negatively correlated with temperature and relative humidity; and positively correlated with rainfall and NDVI (Fig 2). The highest correlations were found between *C*. *oxystoma* abundance and i) temperature averaged between 10 and 7 days prior the capture event (r (10, 7) = -0.575), ii) mean relative humidity between 21 and 19 days prior a capture event ((21, 19) = -0.495), iii) rainfall averaged over the time period of 12 to 10 days prior capture (r (12, 10) = 0.324) and iv) mean NDVI over 80 days prior the capture event (r (8, 0) = 0.617). *C*. *imicola* abundances were negatively correlated with temperature, relative humidity, rainfall and NDVI. The highest correlations were found between *C*. *imicola* abundances and i) temperature averaged over the time period of 29 to 24 days prior capture (r (29, 24) = -0.601), ii) relative humidity averaged over the preceding 22 to 6 days before capture (r (22, 6) = -0.572), iii) rainfall averaged over the time period of 20 to 8 days prior capture (r (20, 8) = -0.565), and iv) NDVI averaged over the 30 days before capture event (r (3,0) = -0.495).

Overdispersion test applied to the 4 models for each species are presented in Table 1. For *C*. *oxystoma* and *C*. *imicola* data, tests were statistically significative for Poisson regression model (p < 10^−3^), negative binomial model (p = 0.0048 for *C*. *oxystoma* and p = 0.0046 for *C*. *imicola*) and Poisson regression mixed-effect model with random effect at the site of capture (p < 10^−3^). However, for both species, overdispersion test was not significative for PRME-SD (p = 1), indicating that there is no overdispersion in this model.

**Table 1:** Overdispersion test and model validation.

Models	*C*. *oxystoma*					*C*. *imicola*				
	Overdispersion test			Validation		Overdispersion test			Validation	
	*χ* ^2^	Ddl	*P* > *χ* ^2^	RMSE_Training	RMSE_Test	*χ* ^2^	Ddl	*P* > *χ* ^2^	RMSE_Training	RMSE_Test
Poisson	45295.6	49	0.0000	178.31	29.4	26002.1	49	0.0000	232.8	78.73
NB	77.2	48	0.0048	195.91	243.01	77.4	48	0.0046	465.5	282.03
PRME-S	12478.3	48	0.0000	131.36	29.41	12393.3	48	0.0000	214.36	41.39
PRME-SD	3.8	48	1.0000	0.72	0.76	2.1	48	1.0000	0.87	0.89

**Fig 3:**
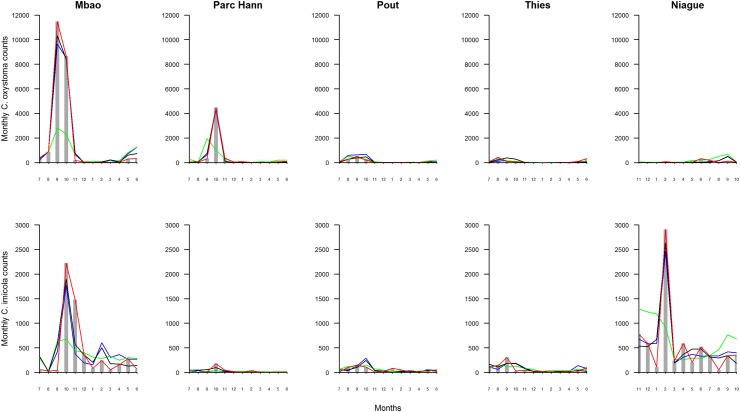
Observed vs Predicted monthly *Culicoides* counts. Monthly *C*. *oxystoma* (A) and *C*. *imicola* (B) counts observed *vs*. predicted by Poisson, NB, PRME-S, PRME-SD, in each site (in column). Months’ quotation: 1 = January to 12 = December.

Results from modelling the female *C*. *oxystoma* and C. *imicola* monthly population dynamics for 4 models are shown in Fig 3. Visual inspection of these results shows that the PRME-SD better estimates the observed data. Further support is provided by the root mean square error (RMSE). PRME-SD models had the lowest mean prediction errors both in the training dataset and in the test dataset: for *C*. *oxystoma* RMSE = 0.72 in the training dataset (respectively RMSE = 0.76 in the test dataset); for *C*. *imicola* RMSE = 0.87 in the training dataset (respectively RMSE = 0.89 in the test dataset) (Table 1).

For each species, covariates that were kept in the final multivariate analysis PRME-SD are presented in Table 2. The number of captured *C*. *oxystoma* increased with the mean temperature at the capture day (Table 2). Relative humidity at the time of capture and from 21 and 19 days prior capture events were positively associated with the *C*. *oxystoma* abundance. High livestock density (values greater than 100 animals per km^2^) increased *C*. *oxystoma* abundance whereas high percentages cover of ‘water bodies’ (values greater than 5%) reduced *C*. *oxystoma* abundance. The interaction between temperature and rainfall had a negative effect on *C*. *oxystoma* abundance. For *C*. *imicola* (Table 2), abundances decreased with the mean rainfall at the date of capture and also with relative humidity between the 22^nd^ and the 6th day before capture events. Opposite, mean NDVI of 10-day covering the capture event and high percentages cover of ‘water bodies’ increased *C*. *imicola* abundance.

**Table 2:** Summary of the PRME-SD abundance model for the two species.

	Regression coefficients	Standard Errors	Z value	P_value
*C*. *oxystoma*				
Intercept	3.8587	0.2378	16.22	< 2e-16
Temperature (mean of capture day)	0.3641	0.0577	6.305	2.85E-10
Humidity (mean of capture day)	0.0563	0.0118	4.739	2.15E-06
H.21.19 (mean humidity from 21 to 19 days prior the capture event)	0.0788	0.0375	2.102	0.0355
Density of ruminants	1.7845	0.5241	3.405	0.0006
Water bodies (percentage cover)	-1.029	0.4849	-2.122	0.0338
Temperature:Rainfall	-0.0084	0.004	-2.095	0.0358
*C*. *imicola*				
Intercept	3.0916	0.1963	15.75	< 2e-16
Rainfall (mean of capture day)	-0.0828	0.037	-2.238	0.0253
NDVI (mean of capture 10-day period)	0.5847	0.1363	4.287	1.81E-05
Water bodies (percentage cover)	2.6365	0.3057	8.623	< 2e-16

## Discussion

McCullagh and Nelder [56] asserted that whenever the variable of interest is a count, its distribution is often an overdispersed Poisson distribution. The present data are another illustration of this assertion. Here overdispersion was not due to excess zeroes because for all sites and during the whole trapping period, *C*. *imicola* was always captured and *C*. *oxystoma* was only absent 1 month at 1 site. Here overdispersion is probably due to very high extreme values. 25% of extremes values represent 79.28% of the total catch for *C*. *oxystoma* and 47.55% for *C*. *imicola*. The first part of this work aimed at comparing which distribution among Poisson, NB, PRME-S and PRME-SD better fitted on counts of *C*. *oxystoma* and *C*. *imicola* recorded using the OVI light traps. For both species, only PRME-SD model dealt with overdispersion and provided the best predictions of the distribution of the observed data.

In the Niayes area of Senegal, both spatial and temporal heterogeneities in *C*. *oxystoma* and *C*. *imicola* abundances were mentioned by Diarra et al. [6]. This can partly be explained by several factors we found associated with these *Culicoides* species.

Abundance of *C*. *oxystoma* was positively correlated to temperature (without any time lag), humidity (with and without time lag) and the density of ruminants, and negatively correlated to the surface of water bodies and to an interaction term of temperature and rainfall. As this work is the first attempt to identify drivers of *C*. *oxystoma*, we will discuss the meanings of these correlations and whether these environmental variables (temperature, humidity, ruminant density, and water body surface) have been involved as drivers of the distribution of other species of *Culicoides*.

Firstly, mean temperature and humidity were positively associated with *C*. *oxystoma* abundance. Indeed, *C*. *oxystoma* peaks were obtained in September-October, at the end of the rainy season and period for which highest temperatures were recorded in our study sites. In Kagoshima, southern Japan, which has a humid sub-tropical climate, this species is also present in very large numbers [45]. It is collected from May to November [45] *i*.*e*. during the warmest and rainiest period of the year, showing that similar seasonal patterns are observed in southern Japan and Senegal. These results also confirm once more the importance of temperature and humidity as major drivers of abundance of many species of insects. Temperature and humidity of capture day can influence adult activity and survival. The correlation between relative humidity from the 21^rst^ to the 19^th^ day prior the collection and adult abundance suggests that it also influences larval and/or pupal stages of *C*. *oxystoma*. More generally, temperature is known to influence greatly the development and survival of all stages of the life cycle of *Culicoides* species [11, 60–66], driving the dynamics and distribution of many *Culicoides* species [16–23, 67]. Concerning humidity, Murray *et al*. [68] showed it was an important criterion for the development and survival of *Culicoides brevitarsis* and Conte *et al*. [25] demonstrated that high relative humidity increased *C*. *imicola* probability of presence in Italy. High livestock densities were positively associated with *C*. *oxystoma* abundance. Presence and numbers of hosts are key factors known to affect catches of other *Culicoides* vector species [46, 69, 70]. High percentage covers of ‘water bodies’ decreased *C*. *oxystoma* abundance. Abundance of *C*. *oxystoma* was negatively associated with the interaction between temperature and rainfall. High rainfall could decrease temperature and inhibit the activity of some *Culicoides* species.

Abundance of *C*. *imicola* was negatively correlated to rainfall, and positively correlated to NDVI and the surface of water bodies. As for *C*. *oxystoma*, *C*. *imicola* populations peaked at the end of the rainy season (September-October period) in the Niayes region [6]. The fact that mean rainfall at the date of capture was negatively associated with *C*. *imicola* abundances could confirm that rainfall can inhibit the activity of some *Culicoides* species as shown by Murray [71]. To a lesser extent, the fact that rainfall can cause *C*. *imicola* nymphs to drown by flooding their breeding sites [72] could also impact the daily catches. Similar results were found by Ducheyne *et al*. [19] for *C*. *imicola* in Spain showing that precipitation, especially summer rainfall (June-September), was the most influential factor determining its distribution. This is concurrent with the observations from Calvete and colleagues [47, 73], who noted that the coefficient of variation and the total amount of precipitation significantly influenced the presence of *C*. *imicola* in Iberia.

In this study *C*. *imicola* abundance was positively influenced by NDVI, a variable correlated with soil moisture [74], precipitation [75] and biomass vegetation [76]. This finding agrees with several other studies which linked high NDVI with increased *C*. *imicola* abundance both in North Africa and in Europe [16, 17, 24, 27, 28, 77]. In Senegal, vegetation growth is important during the rainy season, particularly at the end of the rainy season (September and October), when the highest abundances of *C*. *imicola* were obtained. In Morocco, Baylis et al. [27] suggested that the positive correlation between *C*. *imicola* abundance and average annual minimum NDVI was driven by soil moisture. Finally, results show that high percentages cover of ‘water bodies’ were positively associated with *C*. *imicola* abundance. Moreover, the presence of water bodies in Niague, Mbao and Parc Hann could have provided breeding sites and increased *C*. *imicola* in these sites.

CCM has allowed us to identify time lagged associations between meteorological variables and the abundance of each of the two *Culicoides* species through statistical methods. The use of CCM analysis is a considerable improvement compared to arbitrarily defined time lags. Yet this analysis could be further refined by testing whether the optimal lags found in the CCM vary, seasonally for example, over time. Taking into account eventual seasonal patterns would help better fitting the model throughout the year.

To our knowledge no other published models of *Culicoides* abundance take into account overdispersion. Accounting for overdispersion when it is present is vital; failing to do so can erroneously inflate measures of explained variance [78], bias parameter estimates [79, 80], and may cause researchers to identify variables as having a biologically meaningful effect when in fact they do not [34, 79, 81]. In this work, including random effects for the site and the date enabled to take into account overdispersion, opposite to the three other models tested. The PRME-SD model gave better estimates of *C*. *oxystoma* and *C*. *imicola* abundances: very large differences were noticed between the RMSE of the PRME-SD models and the three others models for both species. For the PRME-SD models, RMSE values are close to zero, and can thus be considered as good. The disadvantage of PRME-SD model is that we cannot predict *C*. *oxystoma* and *C*. *imicola* abundances at other sites. The fact that overdispersion persisted in the other models could indicate that some important variables were omitted or were not included at the correct spatial or temporal resolution. Other variables such as soil moisture [24, 27, 82], wind speed [24, 27, 69, 83–85] and other classes of land cover or landscape metrics [15, 86, 87] could also be explored as they are known to impact *Culicoides* abundance. Modelling *Culicoides* dynamics is very difficult because *Culicoides* catch data is often very “noisy”, with very large variability in catches even at fine spatial [70] or temporal scales and hindered by knowledge gaps on *Culicoides* ecology, as noted by Searle et al. [88], for example. Despite having collected over 220,000 *Culicoides* during this study, more robust models could be obtained with more sites, and/or a finer and/or longer temporal monitoring. Yet larger datasets are scarce because identifying huge numbers of *Culicoides* is difficult and extremely time consuming. To take into account local meteorological variations, temperature and humidity data were collected at a fine scale in each of the study sites. Furthermore CCM enabled to explore the best temporal resolution of the variables tested. This could indicate that climatic variables need to be considered differently, perhaps as degree days for temperature instead of mean daily temperature. Finer spatial resolution of livestock and land cover variables could also help improve the models. Finally, as water bodies seemed to influence the abundance of both species, more attention should be given to better characterizing the larval habitats of this two species in the Niayes area. Modelling *Culicoides* dynamics is an essential step for evaluating the risk of disease transmission as shown by Brugger et al. [55], Guis et al. [89] and Hartemink et al. [90]. Indeed, vector dynamics are necessary to estimate the vector to host ratio, one of the key parameters of R_0_, the basic reproduction ratio, which measures disease transmission risk.

## Conclusions

This study is the first to model the dynamics of *C*. *oxystoma*. It is also the first to investigate the influence of environmental and climatic parameters on the dynamics of *C*. *imicola* in West Africa. This work confirmed that *Culicoides* count data can be extremely variable even at fine spatial and temporal scales. This variability resulted in strong overdispersion of the data. Despite modelling large *Culicoides* counts (over 220,000 *Culicoides* captured in 354 night-traps), using on-site climate measures instead of satellite-derived measures, determining the time-lags for which environmental variables and abundance data were the most correlated through a CCM analysis, overdispersion persisted in all models except the PRME-SD (with nested random effects at the site and date of capture). According to this model, meteorological and environmental variables that contribute to explaining the dynamics of *C*. *oxystoma* and *C*. *imicola* abundances were identified. To take into account overdispersion without including random effects in the models, other variables such as soil moisture, wind speed, degree days, land cover or landscape metrics could be tested. Further work should also assess whether the distribution of counts obtained using host-baited traps are less overdispersed.

## Supporting Information

S1 Data Database for *Culicoides Oxystoma* abundance. (XLSX)

S2 Data Database for *Culicoides imicola* abundance. (XLSX)

S1 Table Correlation coefficients for explanatory variables for *C*. *oxystoma*. Variables found to be highly correlated (coefficient of correlation greater than 0.5) with others covariables were not kept for the multivariate analysis.
(DOCX)

S2 Table Correlation coefficients for explanatory variables for *C*. *imicola*. Variables found to be highly correlated (coefficient of correlation greater than 0.5) with others covariables were not kept for the multivariate analysis.
(DOCX)

S1 Text Statistical description for Multivariate Singular Spectrum Analysis. (DOCX)
