# Supplementary material for: Modelling the Abundances of Two Major Culicoides (Diptera: Ceratopogonidae) Species in the Niayes Area of Senegal
Source: PLoS One. 2015 Jun 29;10(6):e0131021. doi: 10.1371/journal.pone.0131021 (PMC4487250; doi:10.1371/journal.pone.0131021)
Supplement: S1 Table — Variables found to be highly correlated (coefficient of correlation greater than 0.5) with others covariables were not kept for the multivariate analysis. (DOCX) [file pone.0131021.s003.docx]

**Table S1: Correlation coefficients for explanatory variables for *C. oxystoma***

Variables found to be highly correlated (coefficient of correlation greater than 0.5) with others covariables were not kept for the multivariate analysis.

|  | T_mean | H_mean | R_mean | NDVI | T.10.7 | H.21.19 | P.12.10 | N.8.0 |
| --- | --- | --- | --- | --- | --- | --- | --- | --- |
| T_mean | 1 |  |  |  |  |  |  |  |
| H_mean | 0.19 | 1 |  |  |  |  |  |  |
| R_mean | 0.07 | 0.18 | 1 |  |  |  |  |  |
| NDVI | 0.41 | 0.01 | -0.07 | 1 |  |  |  |  |
| T.10.7 | 0.04 | -0.17 | -0.02 | 0.39 | 1 |  |  |  |
| H.21.19 | -0.22 | 0.31 | 0.13 | -0.50 | -0.36 | 1 |  |  |
| P.12.10 | 0.17 | 0.09 | -0.02 | 0.06 | 0.06 | 0.06 | 1 |  |
| N.8.0 | 0.33 | -0.05 | -0.11 | 0.64 | 0.58 | -0.64 | -0.25 | 1 |

T_mean: mean temperature of the capture day, H_mean: mean humidity of the capture day, R_mean: mean rainfall of the capture day, NDVI: mean NDVI of 10-day period including capture day, T.10.7: mean temperature from 10 to 7 days prior the capture event, H.21.19: mean humidity from 21 to 19 days prior the capture event, P.12.10: mean temperature from 12 to 10 days prior the capture event and N.8.0: mean NDVI over 80 days prior the capture event.
