# Supplementary material for: Modelling the Abundances of Two Major Culicoides (Diptera: Ceratopogonidae) Species in the Niayes Area of Senegal
Source: PLoS One. 2015 Jun 29;10(6):e0131021. doi: 10.1371/journal.pone.0131021 (PMC4487250; doi:10.1371/journal.pone.0131021)
Supplement: S2 Table — Variables found to be highly correlated (coefficient of correlation greater than 0.5) with others covariables were not kept for the multivariate analysis. (DOCX) [file pone.0131021.s004.docx]

**Table S2: Correlation coefficients for explanatory variables for *C. imicola***

Variables found to be highly correlated (coefficient of correlation greater than 0.5) with others covariables were not kept for the multivariate analysis.

|  | T_mean | H_mean | R_mean | NDVI | T.29.24 | H.22.6 | P.20.8 | N.3.0 |
| --- | --- | --- | --- | --- | --- | --- | --- | --- |
| T_mean | 1 |  |  |  |  |  |  |  |
| H_mean | 0.14 | 1 |  |  |  |  |  |  |
| R_mean | 0.13 | 0.20 | 1 |  |  |  |  |  |
| NDVI | 0.41 | -0.07 | 0.03 | 1 |  |  |  |  |
| T.29.24 | 0.21 | 0.15 | -0.08 | 0.28 | 1 |  |  |  |
| H.22.6 | -0.10 | 0.34 | 0.05 | -0.68 | -0.28 | 1 |  |  |
| P.20.8 | -0.21 | -0.03 | -0.07 | -0.08 | 0.35 | 0.20 | 1 |  |
| N.3.0 | 0.15 | 0.05 | -0.10 | 0.41 | 0.78 | -0.48 | -0.10 | 1 |

T_mean: mean temperature of the capture day, H_mean: mean humidity of the capture day, R_mean: mean rainfall of the capture day, NDVI: mean NDVI of 10-day period including capture day, T.29.24: mean temperature from 29 to 24 days prior the capture event, H.22.6: mean humidity from 22 to 6 days prior the capture event, P.20.8: mean temperature from 20 to 8 days prior the capture event and N.3.0: mean NDVI over 30 days prior the capture event.
