# Supplementary material for: Modelling the Abundances of Two Major Culicoides (Diptera: Ceratopogonidae) Species in the Niayes Area of Senegal
Source: PLoS One. 2015 Jun 29;10(6):e0131021. doi: 10.1371/journal.pone.0131021 (PMC4487250; doi:10.1371/journal.pone.0131021)
Supplement: S1 Text — (DOCX) [file pone.0131021.s005.docx]

**Text S1**

**Statistical description for Multivariate Singular Spectrum Analysis (MSSA)**

According to Ghil *et al*. [[1](#_ENREF_1)], Singular Spectrum Analysis (SSA) is a data-adaptive, nonparametric method based on embedding a time series $\left\{X\left(t\right):t=1,\ldots,N\right\}$ in a vector space of dimension $M$. The SSA method proceeds by diagonalizing the $M\text{x}M$lag-covariance matrix $C_{X}$of $X(t)$ to obtain spectral information on the time series [[2](#_ENREF_2),[3](#_ENREF_3)]. The matrix $C_{X}$ can be estimated directly from the data as a Toeplitz matrix with constant diagonals, i.e., its entries $C_{ij}$ depend only on the lag $| i-j|$ [[4](#_ENREF_4)], for $1\leq i, j\leq N$:

$$C_{ij}= \frac{1}{N-|i-j|} \sum_{t=1}^{N-|i-j|} X\left(t\right)X(t+|i-j|)$$

Broomhead and King [[4](#_ENREF_4)] proposed computing $C_{X}$by using the $N'\text{ x }M$ *trajectory matrix* $D$ that is formed by $M$lag shifted copies of $X(t)$, which are $N'=N-M+1$ long; then

$$C_{X }= \frac{1}{N'} D^{t}D$$

The eigenvectors $E_{k}$ of lag-covariance matrix $C_{X}$have been called temporal empirical orthogonal functions (EOFs) by Fraedrich [[2](#_ENREF_2)] and by Vautard and Ghil [[3](#_ENREF_3)]. The eigenvalues $\lambda_{k}$of $C_{X}$ account for the partial variance in the direction $E_{k}$ and the sum of the eigenvalues, i.e., the trace of $C_{X}$, gives the total variance of the original time series $X(t)$. Projecting the time series on each EOF yields the corresponding principal components (PCs) $A_{k}$:

$$A_{k}\left(t\right)=\sum_{j=1}^{M} X\left(t+j-1\right)E_{k}(j)$$

An oscillatory mode is characterized by a pair of nearly equal SSA eigenvalues and associated PCs that are in approximate phase quadrature [[1](#_ENREF_1)]. Such a pair can represent efficiently a nonlinear harmonic oscillation. This is due to the fact that a single pair of data-adaptive SSA eigenmodes will often better capture the basic periodicity of an oscillatory mode than methods with fixed [basis functions](http://en.wikipedia.org/wiki/Basis_function), such as the [sines](http://en.wikipedia.org/wiki/Sine) and [cosines](http://en.wikipedia.org/wiki/Cosine) used in the [Fourier transform](http://en.wikipedia.org/wiki/Fourier_transform). The window width$M$ determines the longest periodicity captured by SSA. A Monte-Carlo test can be applied to ascertain the statistical significance of the oscillatory pairs detected by SSA. The entire time series or parts of it that correspond to trends, oscillatory modes or noise can be reconstructed by using linear combinations of the PCs and EOFs, which provide the reconstructed components $R_{K}$.

Here $K$ is the set of EOFs on which the reconstruction is based. The values of the normalization factor $M_{t}$, as well as of the lower and upper bound of summation $L_{t}$and $U_{t}$, differ between the central part of the time series and its endpoints.

**References**

1. Ghil M, Allen MR, Dettinger MD, Ide K, Kondrashov D, et al. (2002) Advanced spectral methods for climatic time series. Rev Geophys 40: 3.1–3.41.

2. Fraedrich K (1986) Estimating dimensions of weather and climate attractors. J Atmos Sci 43: 419–432.

3. Vautard R, Ghil M (1989) Singular spectrum analysis in nonlinear dynamics, with applications to paleoclimatic time series. Physica D 35: 395–424.

4. Broomhead DS, King GP (1986a) Extracting qualitative dynamics from experimental data. Physica D 20: 217–236.
